# Differences in Stylet Sheath Occurrence and the Fibrous Ring (Sclerenchyma) between x*Citroncirus* Plants Relatively Resistant or Susceptible to Adults of the Asian Citrus Psyllid *Diaphorina citri* (Hemiptera: Liviidae)

**DOI:** 10.1371/journal.pone.0110919

**Published:** 2014-10-24

**Authors:** El-Desouky Ammar, Matthew L. Richardson, Zaid Abdo, David G. Hall, Robert G. Shatters

**Affiliations:** 1 United States Department of Agriculture-Agricultural Research Service, Horticultural Research Laboratory, Fort Pierce, Florida, United States of America; 2 United States Department of Agriculture-Agricultural Research Service, South Atlantic Area, Athens, Georgia, United States of America; Louisiana State University & LSU AgCenter, United States of America

## Abstract

The Asian citrus psyllid (ACP, *Diaphorina citri*, Hemiptera: Liviidae), is the principal vector of the phloem-limited bacteria strongly associated with huanglongbing (HLB), the world’s most serious disease of citrus. Host plant resistance may provide an environmentally safe and sustainable method of controlling ACP and/or HLB. Two x*Citroncirus* accessions (hybrids of *Poncirus trifoliata* and *Citrus* spp.), that are relatively resistant (UN-3881) or relatively susceptible (Troyer-1459) to ACP adults with regard to adult longevity, were compared in relation to ACP feeding behavior and some structural features of the leaf midrib. The settling (putative feeding/probing) sites of ACP adults on various parts of the leaf were not influenced primarily by plant accession. However, fewer ACP stylet sheaths were found in the midrib and fewer stylet sheath termini reached the vascular bundle (phloem and/or xylem) in UN-3881 compared to Troyer-1459 plants. Furthermore, in midribs of UN-3881 leaves the fibrous ring (sclerenchyma) around the phloem was significantly wider (thicker) compared to that in midribs of Troyer-1459 leaves. Our data indicate that feeding and/or probing by ACP adults into the vascular bundle is less frequent in the more resistant (UN-3881) than in the more susceptible (Troyer-1459) accessions. Our results also suggest that the thickness of the fibrous ring may be a barrier to stylet penetration into the vascular bundle, which is important for successful ACP feeding on the phloem and for transmitting HLB-associated bacteria. These results may help in the development of citrus plants resistant to ACP, which in turn could halt or slow the spread of the HLB-associated bacteria by this vector.

## Introduction

The Asian citrus psyllid (ACP), *Diaphorina citri* Kuwayama (Hemiptera: Liviidae), is an invasive insect species that transmits the phloem-limited bacteria (*Candidatus* Liberibacter spp.) strongly implicated as the causative agents of huanglongbing (HLB, or citrus greening), the most serious disease of citrus worldwide [1], [2], [3]. ACP and HLB apparently originated in Asia, but one or both are currently distributed in Florida, other parts of the Southern United States, the Caribbean, Mexico, Central and South America, Africa, and several countries in South Asia and the Middle East [1], [3], [4]. In both Brazil and Florida, the disease has spread rapidly throughout commercial and residential citrus plantings causing considerable losses to the citrus industry [3], [4]. Depending on tree age at inoculation with HLB bacteria and other factors, citrus yield is reduced and fruit quality degrades; yield reduction is mainly due to early abortion of fruits from affected branches and can reach 30 to 100% [3]. All commercial citrus varieties are susceptible to HLB disease [1]–[4]. The HLB-associated bacteria in Florida and Asia, *Candidatus* Liberibacter asiaticus (LAS) is known to be transmitted by ACP in a persistent, probably propagative, manner, and thus was found in many tissues of this vector including the gut, salivary glands and hemolymph [2], [3], [4].

Following the discovery of HLB in Florida in 2005, a three-component management program against HLB was advocated initially and implemented by some citrus growers: intensive chemical control of ACP, removal of HLB-infected trees, and planting disease-free nursery stock [3], [4]. However, intensive use of insecticides against ACP is expensive, disruptive to beneficial parasitoids and predators, and not sustainable. Intensive chemical control of ACP can also be ineffective in preventing the introduction and spread of HLB/LAS in new citrus plantings [5]. Thus, development of more effective measures for management of ACP and HLB is of critical importance to ensure the sustainability of commercial citrus in Florida, Brazil and other citrus growing areas where the disease is spreading.

Host plant resistance to ACP, i.e. prevention of colonization or multiplication of ACP on a plant, ultimately may provide the most effective, economical, environmentally safe, and sustainable method of controlling ACP, especially if the plant also is resistant to HLB or other important pests of citrus [3], [4], [6], [7]. Although little resistance to HLB/LAS is found within commercial citrus varieties, tolerance has been reported for some genotypes that are commonly used as rootstocks, notably *Poncirus trifoliata* L. [8], [9], [10]. The ACP has a wide host range within the family Rutaceae, but it reproduces on a narrower range of plants within the subfamily Aurantioideae (citrus subfamily) [1]. Very low numbers of ACP were found on two accessions of *P. trifoliata* in a field survey [11]. *P. trifoliata* is a trifoliate species that is used as rootstock in many citrus-growing regions. It is cross-compatible with *Citrus* species and thus has been an important parent in the development of new rootstocks and scions through intergeneric hybridization with *Citrus* [11]. Nearly all accessions of *P. trifoliata*, and many accessions of x*Citroncirus* spp. (hybrids of *P. trifoliata* and other parent species), were found to reduce oviposition and lifespan of ACP in no choice tests [6]. *P. trifoliata* appears to have antixenosis (deters or prevents colonization) and/or antibiosis (toxicity) in relation to ACP [6], [12]. However, the traits that promote ACP resistance and lower the incidence of HLB have not been identified [6].

ACP and other phytophagous hemipteran insects produce salivary secretions during their feeding or probing. Some of these salivary secretions solidify around the stylets and are termed ‘salivary sheaths’, ‘stylet sheaths’ or ‘stylet tracks’ because, after an insect withdraws its mouthparts, these sheaths remain in plant tissues showing exactly where the insect had been probing and/or feeding [13], [14], [15]. Since the HLB-associated bacterium (LAS) is thought to reside mainly in the phloem of citrus plants, it is likely that LAS transmission cannot be accomplished unless ACP reaches the phloem in order to be able to acquire the bacteria from diseased plants and/or to inoculate it into healthy ones [13]. Using sweet orange leaves [*Citrus sinensis* (L.) Osbeck var. ‘Ridge Pineapple’ or ‘Valencia’] (susceptible to ACP and LAS), we previously demonstrated that the majority (80–90%) of the stylet sheaths termini produced by ACP adults or nymphs that reached a vascular bundle were associated with the phloem whereas only 10–20% were associated with xylem vessels [14]. We also reported that the thick-walled fibrous ring (sclerenchyma layers) around the phloem in sweet orange leaves is more prominent in older than in younger citrus leaves and in the midrib than in smaller veins, and suggested that this fibrous ring may be a barrier to stylet penetration by ACP into the phloem in older leaves [14].

In the present work, we tested the hypothesis that the fibrous ring is thicker, and that ACP stylet sheath occurrence and termini are different, in leaves of two x*Citroncirus* accessions: one is relatively resistant (UN-3881) and the other is relatively susceptible (Troyer-1459) to ACP adults with regard to adult longevity. Mean life span of ACP adults maintained on UN-3881 plants was shown previously to be significantly shorter (nearly one third) that of ACP adults maintained on Troyer-1459 although egg laying was similarly reduced in both of these accessions compared to control plants (*Citrus macrophylla* Wester) [6]. Thus, it should be noted that our investigation is only related to resistance of the two tested x*Citroncirus* accessions as far as ACP adult longevity/life span is concerned, but not to other aspects of resistance (e.g. egg laying or nymphal development).

## Materials and Methods

### Settling/Putative Feeding or Probing Sites of ACP Adults

Young adults of ACP, within one week of eclosion from the last nymphal instar, were taken from a healthy (non-HLB infected) laboratory colony established during the year 2000 that has been maintained on healthy orange jasmine [*Murraya paniculata* (L.) Jack] or citrus trees (*Citrus macrophylla* Wester) in a greenhouse as described by Hall et al. [16]. Seeds of the relatively susceptible (Troyer-1459) and relatively resistant (Unnamed, UN-3881) accessions of x*Citroncirus* were obtained from the USDA-ARS National Clonal Germplasm Repository for Citrus and Dates located at the University of California at Riverside (http://www.citrusvariety.ucr.edu/. In one experiment, ACP-resistant *Poncirus trifoliata* (3330) plants were also used. The numbers associated with these accessions are those given by the Citrus Research Center (CRC). Seeds were planted and grown in a greenhouse (with daily averages of 25–29°C) as previously described [6].

To study ACP settling behavior (putative feeding/probing sites), five younger trifoliate leaves and five older trifoliate leaves (determined based on their position on the branch as well as their color, size, and texture) were detached from plants of the two accessions: UN-3881 and Troyer-1459. The cut-end petioles were placed in small (0.5 ml) microfuge tubes filled with water, and capped with a piece of Parafilm membrane. Each leaf was then placed in a tightly-covered plastic Petri dish along with 5 ACP adults. The dishes were positioned vertically, so that ACP adults could reach both sides of the leaf as previously described [14], and placed on a bench top at 23.7+1.5°C with 14 h light per day. ACP adults that settled (i.e. assumed the typical probing/feeding position, with their body angled about 45° with the leaf surface) were counted twice daily (at 8–9 am and 3–4 pm). During each observation we recorded the number of ACP settling/feeding on the midrib (midvein) and on smaller veins on the adaxial and abaxial sides of the leaf. Counts were also made of ACP adults that were not on the leaf (including live and dead adults). These observations continued for five days, and the experiment was repeated twice, in December 2012 (Expt. 1a) and in January 2013 (Expt. 1b).

### Structural Features of the Midrib and Stylet Sheath Occurrence

**Figure 1:**
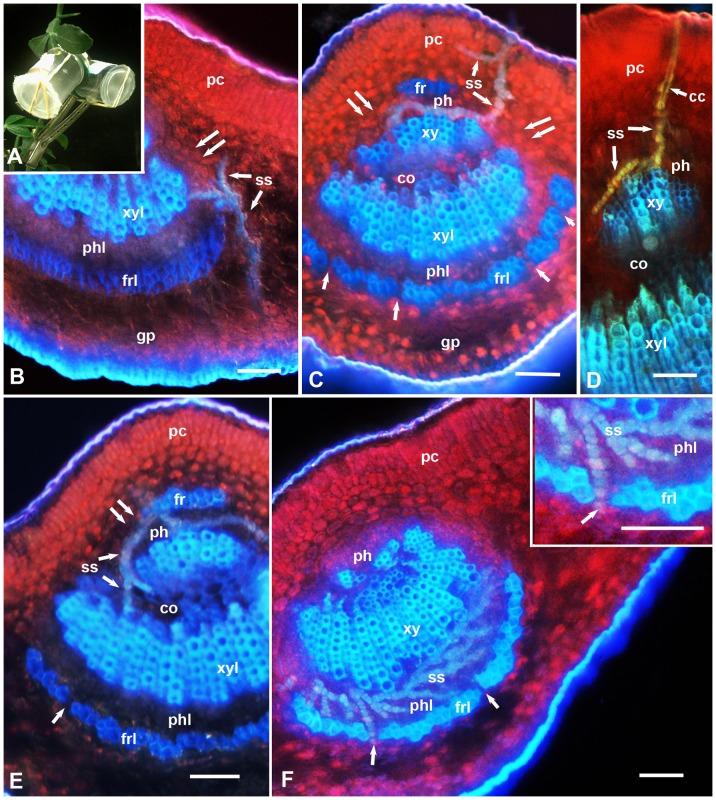
A, Clip cages used for confining adults of *D. citri* on x*Citroncirus* leaves. B–F, Epifluorescence micrographs showing stylet sheaths (ss) of *D. citri* in cross sections of the midrib in leaves of the relatively susceptible (Troyer-1459) plants. In B–F, the upper (adaxial) leaf side is up, and the lower (abaxial) side is down; unlabeled single arrows indicate smaller gaps in the fibrous ring; double arrows indicate wider gaps in this ring at the sides of the vascular bundle. Abbreviations: cc, central canal in the stylet sheath; co, core cells; fr, upper fibrous ring; frl, lower fibrous ring; gp, ground/spongy parenchyma; pc, palisade parenchyma cells; ph, upper phloem; phl, lower phloem; ss, stylet sheath; xy, upper xylem; xyl, lower xylem. Scale bars = 50 µm.

Comparisons were made between Troyer-1459 and UN-3881 plants with respect to leaf midrib structure and ACP stylet sheath occurrence using clip cages to confine ACP adults on young leaves of each plant. The clip cages used (Fig. 1A) were made from plastic tubes, 27 mm long and an inside diameter of 22 mm, with a screen mesh on one end and a plastic cap lined with a felt pad on the other end. The basal part of the central leaflet of a young flush shoot of Troyer-1459 or UN-3881 accessions (comparable in size and position on the plant), was placed in the clip cage, which was secured with a hair clip and a rubber band to insure that no ACP escaped during the experiment. In this experiment, repeated twice, four clip cages were set up on each of the resistant and susceptible accessions: 2 plants from each accession in each of Expts. 2a (January 2013) and 2b (June 2013). In experiment 2a, five ACP adults were confined per clip cage for 5 days, with adults feeding on the upper (adaxial) side of the leaf. In experiment 2b, eight adults were confined per clip cage for 3 days, with 2 cages on the upper (adaxial) side, and 2 on the lower (abaxial) side.

After removing adult ACP from each clip cage, the midrib from the entire leaf area that was inside the cage (exposed to ACP feeding) was cut out longitudinally on both sides with a sharp razor blade. In experiment 2a, the midrib was then cut into four pieces nearly equal in length. Each piece was immediately transferred to a drop of phosphate buffered saline (PBS, pH 7.4; Polysciences) on a clean glass slide, and sectioned by hand using a new clean razor blade to the thinnest possible sections under a stereomicroscope (at 20x or higher). These sections (determined by confocal microscopy to be ca. 50–60 µm thick) were quickly transferred to a fixative solution (4% paraformaldehyde in PBS) for 1–3 days, before washing 3 times with PBS-T (PBS with 0.1% TritonX100). Sections were then stained for 5 min in 3 nM solution of the fluorescent nucleic acid stain propidium iodide, before washing again in PBS-T. In experiment 2b, however, a simpler method was used, in which the midrib pieces were fixed and washed before sectioning, and the sections were not stained. This latter method was adequate when sections were examined only by epifluorescence microscopy, whereas staining in propidium iodide was necessary when using confocal laser scanning microscopy (CLSM) [14,17). In both cases, all the sections obtained from the whole midrib area under the clip cage were processed, mounted on microscope slides using Fluoro-Gel (antifade mounting medium, Electron Microscopy Sciences, Hatfield, PA, USA), and examined. Mounted sections were kept in the dark at 4°C until examination with UV light using an epifluorescence inverted microscope (Olympus IX70, with 4X, 10X or 20X objectives) fitted with a camera system. These sections were examined using autofluorescence (with no filter cubes). Some sections (stained with propidium iodide) were also examined with CLSM (Zeiss LSM 510, 10x or 20x objectives, He/Ne laser) with excitation wavelength of 543, sometimes simultaneously with differential interference contrast (DIC) in order to show cell boundaries.

Morphometrics of the leaf midrib, including width of the phloem and the fibrous ring (sclerenchyma), as well as diameters of the vascular bundle and midrib, were determined from photographed images of these sections using the computer program ‘ImageJ’ “http://rsb.info.nih.gov/ij/index.html”.

### Statistical Analysis

We used a model selection framework using likelihood ratio to evaluate differences in settling (putative feeding or probing) sites of ACP adults on various locations of the leaf in the relatively resistant and susceptible accessions (Table 1). This strategy was used to compare differences between plant accessions (UN-3881 or Troyer-1459), leaves of different age (young or old) and time of day (am vs. pm). The analysis was performed for each experiment separately. This analysis was conducted using the function ‘multinom’ within package ‘nnet’ in R [18], [19]. We conducted stepwise comparisons using likelihood ratio testing between 8 models starting from the simplest and propagating towards the most complex. Only nested models were compared. These models are: 1) a null model (m1), 2) plant alone (accession) model (m2), 3) leaf alone (m3), 4) time alone (m4), 5) plant + leaf (m5), 6) plant + time (m6), 7) leaf + time (m7), and 8) plant + leaf + time (m8). The model showing most significant difference from the null (measured by the p-value resulting from the likelihood ratio test and by comparing the Akaike Information Criteria [AIC]) was chosen to be better fit than the others and kept for the next step. If none of these models showed improvement on the null then the null would be chosen as best fitting the data and we would conclude that the settling behavior on the leaf is independent from the putative effect of plant type, leaf age and time of day. In the second step, the model chosen in the first step is compared to those models (m5, m6, and/or m7) that nest that model. The model that shows the best improvement in fitting the data compared to that from the first step, if any, is kept for the last step. Finally, the model chosen in the second step is compared to the full model (m8) to assess if all explanatory variables influence the settling behavior of these insects (Tables 1, 2, 3).

**Table 1:** Model comparisons to identify the effects that best explain *D. citri* settling behavior data using likelihood testing.

Step	Models Compared	Expt. 1a			Expt. 1b		
		*df*	*LR*	*P value*	*df*	*LR*	*P value*
1	m1 vs m2	4	9.18	0.057	**4**	**25.13**	4.7 * 10^−5^
	m1 vs m3	**4**	**49.56**	4.5 * 10^−10^	4	19.42	6.5 * 10^−4^
	m1 vs m4	4	9.27	0.055	4	4.30	0.37
2	m2 vs m5				**4**	**19.52**	6.2 * 10^−4^
	m2 vs m6				4	4.31	0.37
	m3 vs m5	4	8.26	0.083			
	m3 vs m7	4	9.15	0.057			
3	m5 vs m8				4	4.38	0.36

Models compared are: 1) a null model (m1), 2) plant accession alone model (m2), 3) leaf age alone (m3), 4) time of day alone (m4), 5) accession + leaf age (m5), 6) accession + time of day (m6), 7) leaf age + time of day (m7), and 8) accession + leaf age + time of day (m8); only nested models were compared at each step and significant differences are highlighted using boldface font^1^.

1 *df, LR and P value* refer to the degrees of freedom, likelihood ratio statistic (approximately chi square distributed) and the *P* value of the likelihood ratio test, respectively.

**Table 2:** Proportions of *D. citri* adults, per-settling site, on old and young leaves of x*Citroncirus* plants based on the chosen model (no. 3 in Table 1) that indicated that differences in settling patterns were only associated with the age of the leaf (Expt. 1a).

Leaf age	Adaxial mid-vein	Adaxial smaller veins	Abaxial mid-vein	Abaxial smaller veins	Not on leaf
Old	0.068^a^	0.124^a^	0.453^a^	0.139^b^	0.216^b^
Young	0.032^a^	0.052^b^	0.335^b^	0.268^a^	0.313^a^

Means in the same column followed by a different letter are significantly different.

**Table 3:** Proportions of *D. citri* adults, per-settling site, on leaves of Troyer-1459 or UN-3881 plants based on chosen model (no. 5 in Table 1) that indicated that differences in settling patterns were associated with the interaction between plant accession and leaf age (Expt. 1b).

Plant accession	Leaf age	Adaxial mid-vein	Adaxial smaller veins	Abaxial mid-vein	Abaxial smaller veins	Not on leaf
Troyer-1459	Old	0.176^a,b^	0.124^b^	0.176^b,c^	0.214^a^	0.310^a^
Troyer-1459	Young	0.236^a^	0.093^b^	0.281^a^	0.152^a^	0.238^a,b^
UN-3881	Old	0.133^b^	0.281^a^	0.150^c^	0.186^a^	0.250^a,b^
UN-3881	Young	0.187^a,b^	0.221^a^	0.250^a,b^	0.140^a^	0.202^b^

Means in the same column followed by a different letter are significantly different.

Analysis of variance (ANOVA) and Tukey’s studentized HSD test (SAS Institute, 2010) were used to test differences in the width/thickness of the fibrous ring and other structures in the midrib. Additionally, Chi-square analysis was used to test differences in the occurrence of stylet sheath and their termini between resistant and susceptible plants.

## Results

### Settling/Putative Feeding or Probing Sites of ACP Adults

The settling (putative feeding/probing) sites of ACP adults on various parts of the leaf were not influenced primarily by plant accession (Tables 1, 2, 3). However, in Expt. 1a, differences in ACP settling sites due to leaf age were found (*P*<0.001). ACP were more likely to feed on adaxial small veins and abaxial mid veins (midribs) in old leaves than in young leaves, and less likely to feed on abaxial small veins in old leaves than young leaves (Tables 1 and 2). In Expt. 1b, ACP settling sites were influenced by the interaction between leaf age and plant accession (*P*<0.001). ACP settled more often on adaxial mid veins in young Troyer leaves than old UN-3881 leaves, adaxial small veins in young or old UN-3881 leaves than in young or old Troyer leaves, and abaxial mid veins in young Troyer leaves than old leaves of Troyer and UN-3881 (Tables1 and 3). There was no difference in ACP mortality during the five day period of each experiment between UN-3881 (0–5%) and Troyer-1459 (2–5%) accessions.

### Morphometric Differences in Midrib Structure

**Figure 2:**
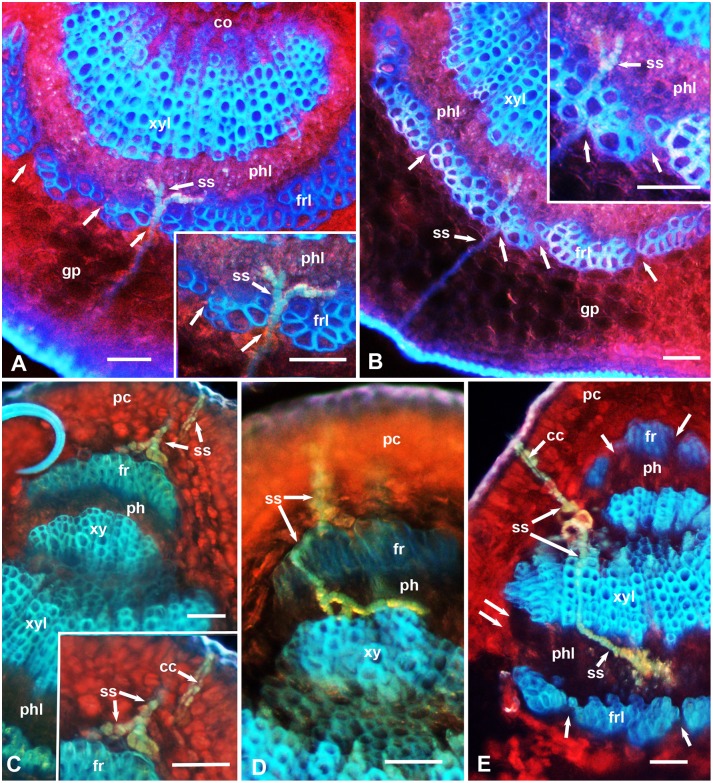
Epifluorescence micrographs showing stylet sheaths (ss) of *D. citri* in cross sections of the midrib in leaves of the relatively resistant (UN-3881) plants. In A–E, the upper (adaxial) leaf side is up, and the lower (abaxial) side is down; unlabeled single arrows indicate smaller gaps in the fibrous ring; double arrows indicate wider gaps in this ring at the sides of the vascular bundle. Abbreviations: cc, central canal in the stylet sheath; co, core cells; fr, upper fibrous ring; frl, lower fibrous ring; gp, ground/spongy parenchyma; pc, palisade parenchyma cells; ph, upper phloem; phl, lower phloem; ss, stylet sheath; xy, upper xylem; xyl, lower xylem. Scale bars = 50 µm.

**Figure 3:**
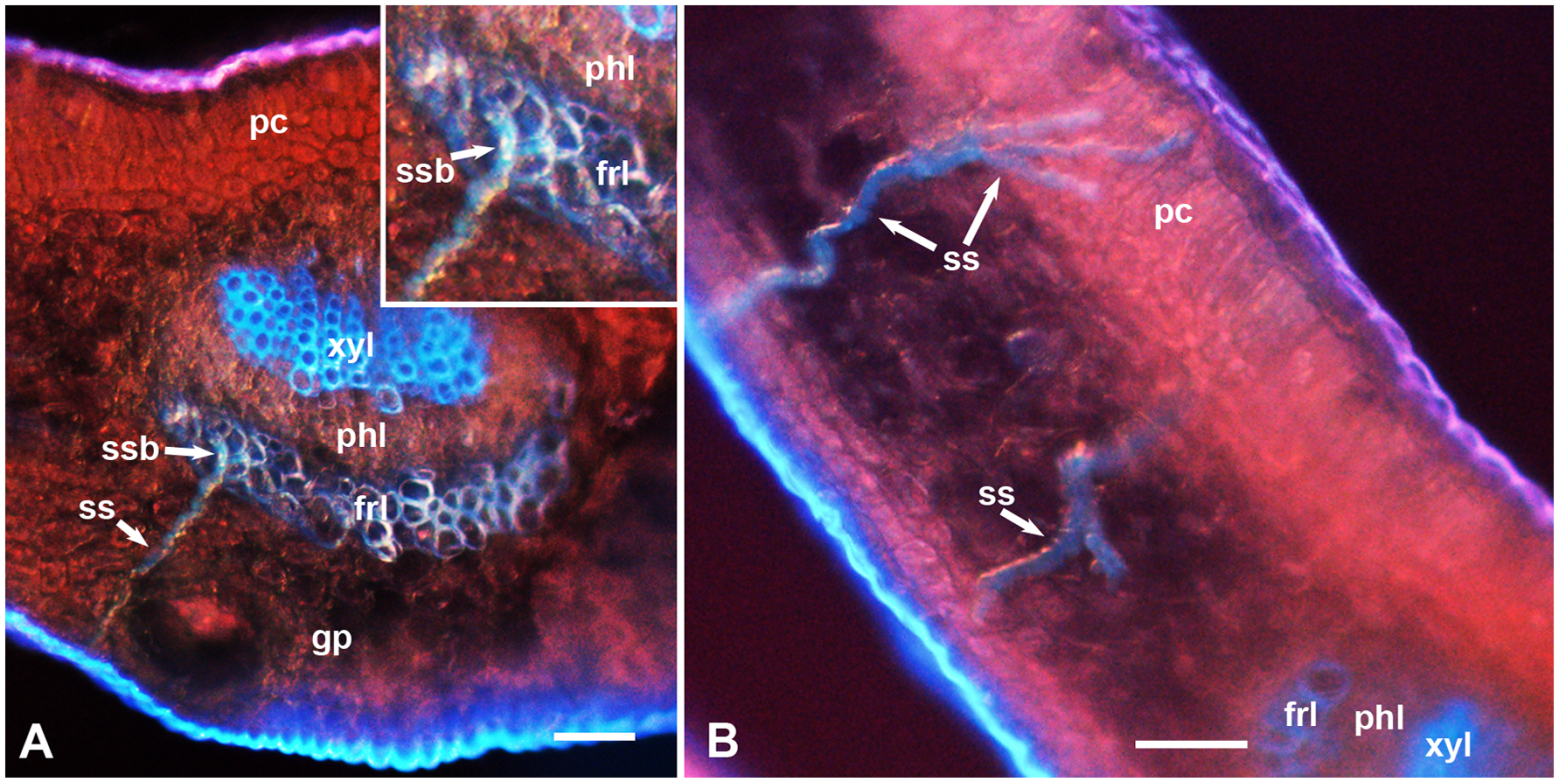
Epifluorescence micrographs showing stylet sheaths (ss) of *D. citri* in cross sections of the midrib (A) or leaf blade (B) of the relatively resistant (UN-3881) plants. Abbreviations: frl, lower fibrous ring; gp, ground/spongy parenchyma; pc, palisade parenchyma cells; phl, lower phloem; ss, stylet sheath; ssb, part of stylet sheath branching inside the fibrous ring; xyl, lower xylem. Scale bars = 50 µm.

**Figure 4:**
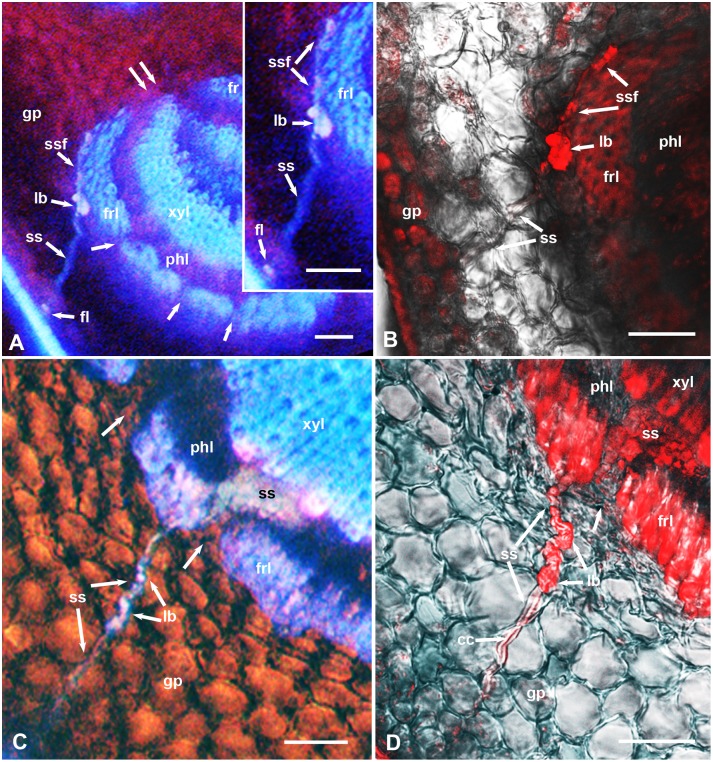
Stylet sheath interactions with the ground parenchyma and fibrous ring in cross sections, stained with propidium iodide, in the midrib of *D. citri*-resistant *Poncirus trifoliata* (A & B) or the relatively susceptible x*Citroncirus* (C & D) plants. A & C, epifluorescence microscopy images; B & D, confocal laser scanning images of the same or adjacent sections as those in A and C, respectively; differential interference contrast was also used in B–D to show cell boundaries. Unlabeled single arrows indicate smaller gaps in the fibrous ring; double arrows indicate wider gaps in this ring at the sides of the vascular bundle. Abbreviations: cc, central canal inside sheath; fl, stylet sheath flange; fr, upper fibrous ring; frl, lower fibrous ring; gp, ground/spongy parenchyma; lb, larger blebs (secretion bursts) in the stylet sheath; pc, palisade parenchyma cells; phl, lower phloem; ss, stylet sheath; ssf, part of stylet sheath going around fibrous ring; xyl, lower xylem. Scale bars = 50 µm.

Epifluorescence microscopy of cross sections in the leaf midrib of x*Citroncirus* plants, that are relatively resistant (UN-3881) or relatively susceptible (Troyer-1459) to ACP adults (with regard to adult longevity), showed that their midrib structure is essentially similar to that described earlier for ACP susceptible *Citrus* plants [14], [20]. Briefly, both the upper (adaxial) and lower (abaxial) epidermis are followed by several mesophyll layers of palisade parenchyma (on the upper side) or ground/spongy parenchyma (on the lower side), followed by the vascular bundle (Figs. 1. 2. 3. 4). In mature leaves, the latter is ensheathed at the upper and lower sides with the fibrous ring (sclerenchyma), which is more prominent on the lower side than on the upper side of the leaf and is discontinuous at both lateral sides of the vascular bundle (Figs. 1 and 2). The upper (adaxial) part of the fibrous ring is rudimentary or absent in the midrib of younger leaves and in smaller veins of young or mature leaves (Figs. 1F, 3A, 3B). The fibrous ring is followed inward by the phloem, xylem, and inner core cells, respectively. Using only autofluorescence with UV light (no filters), the palisade and spongy parenchyma layers appeared in bright red (probably due to the chlorophyll in their chloroplasts), xylem vessels appeared in bright blue, the fibrous ring in dark blue, whereas ACP stylet sheaths appeared usually bluish-yellowish in color (Figs. 1, 2, 3, 4). The phloem at the lower (abaxial) leaf side is normally thicker and much more well-defined than that on the upper side (Figs. 1C–F, 2E). Thus, in the following morphometric results, the ‘phloem width’ refers only to the lower/abaxial part of phloem.

Significant differences in the morphometrics of some structures in the leaf midrib were found between the relatively resistant (UN-3881) and the relatively susceptible (Troyer-1459) plants (Table 4). The width/thickness of both the upper and lower parts of the fibrous ring, whether measured independently or in relation to the diameters of the vascular bundle or the midrib vein, was larger in UN-3881 than in Troyer-1459 plants (P<0.001). The width of the lower/abaxial phloem, whether measured independently or in relation to the diameters of the vascular bundle or the midrib vein, was also larger in UN-3881 than in Troyer-1459 plants (P<0.001). Finally, the diameters of both the vascular bundle and the midrib were larger in UN-3881 than in Troyer-1459 plants (P<0.001).

**Table 4:** Morphometrics of the fibrous ring (FR), phloem, and vascular bundle in leaf midrib sections of the relatively resistant (UN-3881) and relatively susceptible (Troyer-1459) plants^1^.

Parameter	Plant accession	Upper FR	LowerFR	Phloem	Vascular bundlediameter	Vein diameter
Mean width/thickness (µm)	UN-3881	26.4a	28.7a	74.7a	402.7a	665.0a
	Troyer-1459	17.2b	21.2b	52.2b	371.6b	627.0b
% relative to vascular bundle diameter	UN-3881	6.4a	7.4a	18.4a	–	–
	Troyer-1459	4.3b	6.0b	14.2b	–	–
% relative to vein diameter	UN-3881	3.9a	4.4a	11.1a	60.2a	–
	Troyer-1459	2.6b	3.5b	8.4b	59.0a	–

1 No. of midrib sections examined by epifluorescence microscopy were 46 for Troyer-1459 and 47 for UN-3881. For each parameter, means in the same column followed by a different letter are significantly different (*P*<0.001) (Tukey’s studentized HSD test).

### Stylet Sheath Occurrence and Termini

Significant differences were found between the two tested x*Citroncirus* accessions with regard to the occurrence of ACP stylet sheaths in the leaf midrib, and in the proportion of stylet sheath termini found in the vascular bundle (phloem and/or xylem) (Table 5). Stylet sheaths were found in 16.4% of the midrib sections from leaves of the relatively susceptible (Troyer-1459) compared to only 5.1% of those from the relatively resistant (UN-3881) plants (χ^2^ = 26.8, P<0.001**)**. Additionally, the percentage of stylet sheath termini found in the vascular bundle (as opposed to those that were found in the mesophyll) were significantly higher in Troyer-1459 (85.7%) than in UN-3881 (62.1%) (χ^2^ = 5.21, P<0.006). In both accessions, however, the percentage of sheath termini that were found associated with xylem elements (11.1–16.7%) was much lower than that of those found in the phloem (83.3–88.9%), with no significant differences between Troyer-1459 and UN-3881 in this regard. Stylet sheaths were observed to extend or branch from the phloem to the xylem elements apparently either from the inner periphery of the phloem closest to the xylem (Figs. 1B–1D, 2D) or by going from the sides of the vascular bundle through its inner core to inner layers of the xylem elements (Fig. 1E).

**Table 5:** Occurrence of stylet sheaths in leaf midrib sections after using clip cages to confine *D. citri* on leaves of the relatively resistant (UN-3881) or relatively susceptible (Troyer-1459) plants^1^.

Parameters	Plant accession	Expt. No.					
		Expt. 2a		Expt. 2b		Total	
		No.	%	No.	%	No.	%
No. sections with sheaths/total no. sections	UN-3881	10/76	13.2	19/495	3.8	29/571	5.1
	Troyer-1459	51/101	50.5	19/325	5.8	70/426	16.4
No. sheath termini in vascular bundle/total no. sections	UN-3881	6/76	7.9	12/495	2.4	18/571	3.2
	Troyer-1459	50/101	49.5	22/325	6.8	72/426	16.9
No. sheath termini in vascular bundle/total no. sheaths	UN-3881	6/10	60.0	12/19	63.2	18/29	62.1
	Troyer-1459	50/60	83.3	22/24	91.7	72/84	85.7
No. sheath termini in xylem/no of sheaths in vas bundle	UN-3881	1/6	16.7	1/12	8.3	2/18	11.1
	Troyer-1459	9/50	18.00	3/22	13.7	12/72	16.7

1 Five *D. citri* adults were confined/clip cage for 5 days in expt. 2a, and 8 adults confined for 3 days in expt. 2b (4 reps/expt.).

Generally, in both accessions, the stylet sheaths entering the midrib from the upper (adaxial) leaf side were mainly associated with the upper-side phloem (Figs. 1C–1E, 2D), whereas those entering from the lower (abaxial) leaf side were observed to be associated mainly with the lower-side phloem (Figs. 1B, 2A, 2B). In some cases, however, the stylet sheaths entering from the upper leaf side were observed to extend their reach to the lower-side phloem either from the side of the vascular bundle (Fig. 1F) or going through/between xylem elements to the other side of the phloem (Fig. 2E). In Fig. 1F extensive branching of the stylet sheaths can be seen in the lower-side phloem in the leaf of a Troyer-1459 plant although ACP adults were confined to the upper side of the midrib on this leaf. No such extensive branching of stylet sheaths in the phloem was observed in the relatively resistant (UN-3881) plants (Fig. 2).

### Stylet Sheaths Pathways and Their Interaction with the Fibrous Ring and Other Leaf Tissues

In the two x*Citroncirus* accessions studied, the stylet sheaths starting either from the upper (adaxial) or the lower (abaxial) sides of the leaf midrib normally took a slightly tenuous (apparently intercellular) course through the palisade or ground/spongy parenchyma layers before entering the vascular bundle (Figs. 1, 2, 3, 4). Within the ground or palisade parenchyma, the majority of these sheaths seemed to be directed almost straight to the vascular bundle, although in a few cases short sideway branches in the mesophyll parenchyma were found (Fig. 1C). Also, in a few cases, some stylet sheaths starting on the lower side of the leaf blade, some distance away from the midrib, were found to traverse the ground parenchyma straight to branch inside the palisade parenchyma layers near the upper leaf side (Fig. 3B). However, it is not known whether these sheaths actually entered palisade cells or passed between them, and if they missed or entered the smaller veins/vascular bundles running through the leaf blade.

The fibrous ring (sclerenchyma), partially surrounding the phloem in the leaf midrib is composed of 1–5 layers of thick-walled fibers with little or no space between fiber cell walls (Figs. 1F, 2A, 2B, 3A, 4B). However, several narrow channels (gaps), either straight (Figs. 2A, 4C–4D) or crooked (Fig. 2B inset), were observed in both the lower and upper parts of the fibrous ring (on the abaxial and adaxial sides, respectively). Additionally, a much wider gap exists on both (lateral) sides of the vascular bundle between the upper and lower parts of the fibrous ring (Figs. 1, 2, 3, 4). In sections from resistant and susceptible plants, most of the stylet sheaths were observed to enter the vascular bundle either from the wider gaps between the upper and lower parts of the fibrous ring (Figs. 1B, 1C, 2E) or through the smaller gaps in the fibrous ring itself (Figs. 2A, 2B, 2D, 4C–D). In some cases, the stylet sheaths seemed to take an almost straight course through the mesophyll parenchyma into one of these smaller or wider gaps (Figs. 1B, 1C, 2A, 2B). In other cases, the stylet sheath seemed to branch (Fig. 2C) or bend around the fibrous ring sometimes for a relatively long distance (Figs. 2D, 4A), presumably searching for one of these wider or narrower gaps to use for entering the phloem (Fig. 2D). In one case, the stylet sheath appeared to branch inside the fibrous ring (Fig. 3A), probably following one of the narrow crooked gaps inside this ring.

While going through the outer mesophyll parenchyma layers, the stylet sheath is mostly cylindrical with a slightly gnarled (beaded) appearance around a central canal that was previously occupied by the stylet bundle (Figs 1, 2, 3, 4). However, in deeper areas, closer to the fibrous ring and inside the vascular bundle, wider blebs (probably representing larger amounts of salivary secretion bursts) were observed in these parts of the stylet sheath (Figs. 1B–1F, 2A–2C, 4A–4D). On examining midrib sections with epifluorescence microscopy, much brighter autofluorescence was normally observed in deeper and wider areas of the stylet sheath closer to the fibrous ring or inside the vascular bundle compared to parts of the stylet sheath going through the outer layers of the mesophyll parenchyma (Figs. 2B, 4A, 4C). This difference in autofluorescence and/or composition between various areas of the stylet sheath was confirmed with CLSM of propidium-iodide stained sections in both susceptible (x*Citroncirus*) and resistant (*P. trifoliata*) plants (Fig. 4). Using He/Ne laser, with excitation wavelength of 543, the same areas of the stylet sheath (showing brighter autofluorescence) deeper in the parenchyma closer to the fibrous ring, or those inside the vascular bundle, were observed in bright red color by CLSM, whereas the rest of the stylet sheath going through outer layers of the mesophyll were either colorless or much lighter red (Figs. 4A–4D).

## Discussion

Host plant resistance is an essential component of integrated pest management programs for ACP, HLB and other pest/pathogen systems [3]–[6], [8]–[12]. In the present work, we showed that the fibrous ring (sclerenchyma) around the phloem is thicker/wider in the leaf midribs of an accession that is relatively resistant to ACP adults (UN-3881) compared to the fibrous ring in a relatively susceptible accession (Troyer-1459). The difference in thickness of the fibrous ring was significant whether measured independently or in relation to the width of the vascular bundle or the midrib which suggests that this difference is independent of the leaf age or midrib thickness. Furthermore, we showed that the occurrence of ACP stylet sheaths, and the proportion of stylet sheath termini that reached a vascular bundle, were greater in the relatively susceptible than in the relatively resistant accession. This is the first report that correlates the thickness of the fibrous ring with ACP resistance in host plants. We observed no differences in settling (putative feeding or probing) sites of adult ACP due strictly to their relative susceptibility, but susceptibility may interact with leaf age to influence selection of these sites by ACP. However, the occurrence of stylet sheaths, especially those reaching the phloem, is probably a better indicator of plant host acceptability for ACP than settling sites, especially in relation to HLB-pathogen transmission [13], [14], [21]. ACP adult longevity was shown previously to be significantly shorter in UN-3881 than inTroyer-1459 although egg laying was similarly reduced in both of these x*Citroncirus* accessions compared to control plants (*Citrus macrophylla* Wester) [6]. However, citrus plants truly susceptible to all ACP stages (e.g. *C*. *macrophylla*) were not used here because their leaf shape and size are vastly different from the trifoliate leaves of *xCitroncirus* spp., which would make morphometric and structural comparisons between the two types of leaves very difficult and unreliable. Thus, it should be noted that our investigation is only related to resistance of the two tested x*Citroncirus* accessions as far as ACP adult longevity is concerned, but not to other aspects of resistance (e.g. egg laying or nymphal development).

Using Raman microscopy analysis, Richter et al. [22], indicated that the highest lignification in plant cell walls was observed in the sclerenchyma, suggesting that these fibrous layers can act as a barrier for protection of the vascular bundle. That the fibrous ring can also be a barrier to stylet penetration may be indicated by the fact that, in the present work, most of the observed stylet sheaths entered the vascular bundle either through the narrow gaps in the fibrous ring or through the wider gaps in this ring on the lateral sides of the vascular bundle. These gaps have been shown previously to be occupied with thinner-walled cells similar to those of the ground parenchyma [14]. In many cases, ACP seemed to bend their stylets around the fibrous ring in order to enter the vascular bundle (Figs 1, 2, 4). Similarly, the aphid *Megoura viciae* (Buckton) (Hemiptera: Aphididae) was reported to make a stylet ‘detour’ when encountering arches of tough sclerenchyma cells along the phloem in their host plant *Vicia fabae* L. [23]. Although we used only adult ACP in our present work, this should be even more applicable to ACP nymphs, especially younger instars, whose stylets are considerably shorter and thinner (i.e. probably less able to penetrate deeper and tougher tissues) than those of the adults [14]. Since reaching the phloem is important for acquisition and/or inoculation of HLB bacteria by ACP [13], [21] we suggest that this trait (thickness of the fibrous ring) can be used for selection of citrus lines that are resistant to ACP, which in turn can probably halt or slow down the spread of HLB bacteria by this important vector.

In the present work, among ACP stylet sheaths that reached the vascular bundle in both accessions, the great majority of sheath termini reached the phloem while very few were associated with xylem vessels. This is consistent with our previous report on ACP stylet sheaths in *Citrus* [14], and suggests that the relative resistance of UN-3881 to ACP adults is not specifically associated with adult stylets not reaching the phloem, but with them not reaching the vascular bundle at all (neither xylem nor phloem). Our present report is also the first to indicate that the autofluorescence and staining characteristics of the stylet sheath parts closer to the fibrous ring, as well as those inside the vascular bundle, are different from those parts passing through the outer mesophyll parenchyma layers. This may indicate differences in the quantity and/or constitution of the salivary secretions composing that sheath around the stylet bundle as it approaches or passes through tougher cell walls (e.g. sclerenchyma fibers, phloem elements or xylem vessels). Thus, ACP may be producing certain enzymes (or more of them) only in these areas to facilitate its stylet passage through or around these tougher cell walls. Other phloem feeding hemipterans are able to adapt salivary secretions according to their stylet tip milieu. For example, the soluble protein fraction (watery saliva) produced by the aphid *M. viciae* was almost exclusively secreted in sieve elements, whereas the nonsoluble (stylet sheath) fraction was preferentially secreted at cell wall conditions [24].

Using electrical penetration graphs (EPG), Bonani et al. [13] reported that ACP adults started phloem ingestion on young citrus leaves much faster than on mature leaves, and proposed that these differences may be due to a thicker layer of fiber cells (i.e. sclerenchyma). With the Southern chinch bug *Blissus insularis* Barber (Hemiptera: Blissidae), the salivary sheaths in St. Augustinegrass were more abundant on the outermost leaf sheath of axillary shoots of resistant cultivars compared with susceptible ones, suggesting that the thick-walled sclerenchyma cells play a role in chinch bug resistance possibly by reducing stylet penetration into the vascular tissue [25]. However, other factors may contribute to insect resistance in plants even when the stylets have reached the phloem. Nymphs and adults of the European pear psylla (*Cacopsylla pyri* L.) do not ingest from the phloem in resistant selections of pear for any prolonged period, which suggests that resistance factors are located in the phloem sap rather than factors outside the phloem [26]. Factors inside and outside the phloem seem to be involved with resistance to the soybean aphid, *Aphis glycines* Matsumura where EPG studies showed the following: a. soybean aphids feeding on susceptible genotypes had a significantly greater duration of sieve element phase than when feeding on resistant genotypes, b. the time taken to reach the first sieve element phase in resistant genotypes was significantly greater than in susceptible ones, and c. most of the aphids reached sieve element phase in susceptible genotypes but only a few reached this phase in resistant genotypes [27]. The last two findings (b and c) are consistent with our results regarding fewer ACP stylet sheath termini reaching the vascular bundle in resistant vs. susceptible accessions. Other EPG studies on soybean aphids [28] and the leafhopper *Cicadulina mbila* (Naude) [29] indicated that a greater proportion of insects ingested from xylem, rather than the phloem, when feeding on resistant lines, unlike what we found with ACP. With the whitefly *Trialeurodes vaporariorum* (Westwood), resistance factors were associated with phloem ingestion in resistant tomato genotypes, but with other factors (not related to the phloem) in sweet pepper genotypes [30]. The latter factors may include barriers to stylet penetration into the phloem or vascular bundle, including the fibrous ring (sclerenchyma), as we suggested here.

Differences in the feeding behavior of a pathogen vector may affect its ability to acquire and/or inoculate this pathogen from or to its host plant. An EPG study on the aphid *Rhopalosiphum padi* L., vector of the phloem limited barley yellow dwarf virus (BYDV), indicated differences in the phloem salivation and phloem feeding phases between aphids feeding on barley lines resistant or susceptible to BYDV, and suggested that the phloem salivation time was too short for successful infection by BYDV in the resistant lines [31]. Thus, fewer ACP stylet sheaths reaching the phloem in resistant citrus plants may similarly be a hindrance to acquisition and/or inoculation of HLB bacteria. EPG studies on ACP, combined with anatomical studies like ours and with chemical analysis of the phloem sap, should elucidate further the structural, physical and chemical factors that may be responsible for resistance to ACP in non-favorable host plants. Development of resistant citrus plants to ACP and/or HLB is urgently required and may ultimately be the most effective, environmentally safe, and sustainable method of controlling this disease that is currently plaguing and threatening the citrus industry in the US and other parts of the world.
